# lncRNA ZEB1-AS1 Mediates Oxidative Low-Density Lipoprotein-Mediated Endothelial Cells Injury by Post-transcriptional Stabilization of NOD2

**DOI:** 10.3389/fphar.2019.00397

**Published:** 2019-04-16

**Authors:** Xiaohui Xu, Congmin Ma, Zhihui Duan, Yanjiao Du, Chao Liu

**Affiliations:** Department of Neurology, Luoyang Central Hospital Affiliated to Zhengzhou University, Luoyang, China

**Keywords:** endothelial dysfunction, oxidative low-density lipoprotein, ZEB1-AS1, nucleotide-binding oligomerization domain 2, leucine rich pentatricopeptide repeat containing

## Abstract

Oxidized-low density lipoprotein (ox-LDL) can induce injury of endothelial cells, causing atherosclerosis, which is an important initial event in several cardiovascular diseases. Long non-coding RNAs (lncRNAs) have emerged as regulators of diverse biological processes, but their specific biological functions and biochemical mechanisms in ox-LDL-induced endothelial cell injury have not been well investigated. Here, we describe the initial functional analysis of a poorly characterized human lncRNA ZEB1 antisense 1 (ZEB1-AS1). We found that ox-LDL treatment could induce a decreased cell viability and an increased cell apoptosis in endothelial cells, and knockdown of ZEB1-AS1 significantly reversed this effect. Mechanistically, ox-LDL treatment could sequester p53 from binding to ZEB1-AS1 promoter region, causing transcriptional activation and upregulation of ZEB1-AS1. Moreover, enhanced ZEB1-AS1 could upregulate Nucleotide-Binding Oligomerization Domain 2 (NOD2) expression through recruiting leucine-rich pentatricopeptide repeat motif-containing protein (LRPPRC) to stabilize NOD2 mRNA. Experimental data showed that knockdown of NOD2 or LRPPRC dramatically abrogated the functional role of ZEB1-AS1 in ox-LDL-induced endothelial cell injury. In summary, we demonstrated that lncRNA ZEB1-AS1 regulates the ox-LDL-induced endothelial cell injury via an LRPPRC-dependent mRNA stabilization mechanism. Therefore, ZEB1-AS1 may serve as a multi-potency target to overcome endothelial cell injury, atherosclerosis and other cardiovascular diseases.

## Introduction

The dysfunction of endothelial cells (ECs), which is closely related with endothelial damage, play key roles in the initiation and progression of atherosclerosis (AS) (Blann, 1999). AS is the primary factor underlying cardiovascular and cerebrovascular diseases with high morbidity and mortality rates (Kanemitsu et al., 2011). Evidence has indicated that oxidized low-density lipoprotein (ox-LDL), are predominantly engulfed by macrophages, resulting in increased pro-inflammatory cytokine expression and further endothelial cell injury, thus contributing to progression of AS and other cardiovascular diseases (Chen et al., 2002). Although ox-LDL is a critical regulator during ECs injury, the precise regulatory mechanism is still not well known.

Long non-coding RNAs (lncRNAs) are a class of poor conserved endogenous RNAs longer than 200 nucleotides that do not encode proteins but regulate gene expression (Loewen et al., 2014). On a functional level, lncRNAs are involved in complex biological processes through diverse mechanisms. These comprise, among others, gene regulation by titration of transcription factors, alternative splicing, sponging of microRNAs, and recruitment of chromatin modifying enzymes (Kornienko et al., 2013; Fatica and Bozzoni, 2014; Nakagawa and Kageyama, 2014; Ni et al., 2017). Accumulating evidence indicates that lncRNAs play diverse roles in the AS and ECs dysfunction, such as MALAT1 (Gast et al., 2018), ANRIL (Holdt and Teupser, 2018), and H19 (Pan, 2017). Previously, we identified that lncRNA XIST (X-inactive specific transcript) regulates ox-LDL-mediated ECs injury through modulation of miR-320/NOD2 axis (Xu et al., 2018). However, functional potentials of other lncRNAs are waiting to be revealed.

The lncRNA ZEB1-antisense 1 (ZEB1-AS1, transcript ID: ENST00000607166.1) derives from the promoter region of ZEB1, a transcriptional factor that plays important roles in physiology and tumorigenesis (Li et al., 2016). As a famous epithelial-mesenchymal transition (EMT) promoter, ZEB1 plays important roles in ECs transition and cell adhesion (Gu et al., 2015). For example, ZEB1 could transcriptionally promote the expression of intercellular adhesion molecule-1 (ICAM-1), inducing the monocyte-macrophage adhesion and atherosclerotic lesion formation (Abou-Jaoude et al., 2018). However, whether lncRNA ZEB1-AS1 is involved in the ox-LDL-mediated ECs injury and AS, and if so, what is the underlying mechanism is still unknown.

In our previous study, we revealed the essential role of NOD2, which plays important roles in innate immunity by recognizing bacterial lipopolysaccharides (Caruso et al., 2014), in ox-LDL-induced ECs. Here, we sought to identify the functional role of ZEB1-AS1 in ox-LDL-mediated injury of human umbilical vein endothelial cells (HUVECs) and investigated its function by regulating NOD2 and interacting with leucine-rich pentatricopeptide repeat motif-containing protein (LRPPRC), which could promote RNA stability (Siira et al., 2017; Chen et al., 2019). We revealed that knockdown of ZEB1-AS1 alleviated the ox-LDL-induced ECs apoptosis. Mechanistically, ZEB1-AS1 guided LRPPRC to promote the stability of NOD2 mRNA, contributing to increase nucleotide-binding oligomerization domain 2 (NOD2) protein expression. Therefore, targeting ZEB1-AS1 could be a potential therapeutic strategy leading to lessen ECs injury related disease, such as AS.

## Materials and Methods

### Cell Culture and Reagents

HUVECs were purchased from American Tissue Culture Collection (ATCC, Manassas, VA, United States), and were cultured in Dulbecco’s modified Eagle (DMEM, Gibco, Carlsbad, CA, United States) medium with 10% fetal bovine serum (FBS) (Gibco BRL, Grand Island, NY, United States) at 37°C in 5% CO_2_. ox-LDL was purchased from Sigma-Aldrich (St. Louis, MO, United States) and used at the concentration of 100 μg/ml as previously described (Che et al., 2017; Xu et al., 2018).

### Vector Construction and Cell Transduction

Short interfering RNA (siRNA) oligonucleotides targeting ZEB1-AS1, LRPPRC, NOD2 and negative control siRNAs were designed and purchased from GenePharma (Shanghai, China). The siRNA sequences are listed in Table 1. SiRNA transfections were performed using 75 nM siRNA and Lipofectamine RNAimax (Life Technologies, Waltham, MA, United States). The overexpression vector with full-length of ZEB1-AS1 was cloned into plasmids by GenePharma. The sequences for siRNAs were shown in Table 1.

**Table 1:** Information of the qPCR primer sequences and silencing RNA sequences.

qPCR primer name	Sequence (5′–3′)
ZEB1-AS1 (Forward)	TGGCACCCGTGACGACTTAACT
ZEB1-AS1 (Reverse)	GTAGTGGATCGTGTTACTGTGT
NOD2 (Forward)	CCAGCGTCTTTGGCCATTCAACAT
NOD2 (Reverse)	TTGAGCTCATCCAGTGCTTGGAGT
GAPDH (Forward)	GCACCGTCAAGGCTGAGAAC
GAPDH (Reverse)	ATGGTGGTGAAGACGCCAGT
U6 (Forward)	CTCGCTTCGGCAGCACA
U6 (Reverse)	AACGCTTCACGAATTTG
U1 (Forward)	GGGAGATACCATGATCACGAAGGT
U1 (Reverse)	CCACAAATTATGCAGTCGAGTTTCC
Silencing RNA name	Sequence (5′–3′)
si-ZEB1-AS1#1	GGGTGTAAAAGAACCCGTA
si-ZEB1-AS1#2	GAATCATAACCTTTATTGCA
si-ZEB1-AS1#3	GGCACCCTAGGATCCACCGA
si-LRPPRC	GGAGGAGCAUUUGAGATT
si-NOD2	TGATCAGCCTCAATCTGCA
si-NC	GACCTACAACTACCTATCA

### Reverse Transcription (RT) and Quantitative Real-Time Polymerase Chain Reaction (qRT-PCR)

Total RNA was extracted from HUVECs by using the Qiagen RNeasy Mini Kit according to the manufacturer’s instructions (Qiagen, Hilden, Germany). Dnase I was used to eliminate the potential DNA pollutions. and RT and qPCR kits were used to evaluate the expression of target RNAs. RT (20 μl) reactions were performed using the PrimeScript^®^RT reagent kit (Takara, Dalian, China) and incubated for 30 min at 37°C followed by 5 s at 85°C. The quality of RT- samples were controlled by testing the density and purity. The 260/280 values should be between 1.8 and 2.0, and the density should be more than 200 μg/ml. For qPCR, 2 μl of diluted RT product was mixed with 23 μl reaction buffer provided by Takara (Takara Inc., Dalian, China) to a final volume of 25 μl. All reactions were carried out using an Eppendorf Mastercycler EP Gradient S (Eppendorf, Germany) under the following conditions: 95°C for 30 s followed by 45 cycles of 95°C for 5 s and 60°C for 30 s. The transcript expression of glyceraldehyde-3-phosphate dehydrogenase (GAPDH) was used for the normalization of detected RNAs using the comparative 2^−ΔΔCq^ method (Wagner, 2013). The primer sequences for qPCR are presented in Table 1.

### Rapid Amplification of cDNA Ends (RACE)

Both 5′ RACE and 3′ RACE were performed. In 3′ RACE, cDNAs were generated using an Oligo-dT primer that complemented the natural polyA tail of mRNAs. PCR was then used to amplify cDNA product from the 3′ end of ZEB1-AS1 with a sense specific primer and Oligo-dT primer. In 5′ RACE, an antisense gene specific primer was used to produce cDNA from the 5′ end of ZEB1-AS1. Next, a string of identical nucleotides (dATP) were added to the 3′ end of the cDNA. PCR was then carried out to amplify cDNA from the 5′ end using an antisense specific primer and Oligo-dT primer. We used a priming strategy in which both the 5′ and 3′ RACE reactions were primed using the same primer sequence, albeit reverse complemented, to ensure amplification of a contiguous long transcript.

### Cell Viability Assay

The cell viability was assayed using the MTT Kit (Dojindo, Rockville, MD, United States). In brief, cells were seeded into a 96-well plate and then treated with ox-LDL for 24 h at the concentration of 100 μg/ml. After, cells were treated with the MTT reagent and further cultured for 2 h. The optical density at 450 nm was measured with a spectrophotometer (Thermo Electron Corporation, Waltham, MA, United States).

### Flow Cytometry Apoptosis Assay

Cells were collected after ox-LDL treatment or transient transfection for 48 h, then washed with PBS and trypsin containing 0.025% trypsin-EDTA to get the single-cell suspensions. They were then fixed in ice-cold 70% ethanol and with Annexin-V FITC and propidium iodide (PI) solution (Sigma-Aldrich; Merck KGaA, Darmstadt, Germany). Apoptosis was detected by on a BD FACSCalibur instrument.

### TUNEL Assay

For TUNEL assay, cells cultured in chamber slide were fixed with 4% paraformaldehyde for 30 min. Then cells were stained with TUNEL kit on ice according to the manufacturer’s instructions (Vazyme, TUNEL Bright-Red Apoptosis Detection Kit, A113). TUNEL-positive cells were counted under fluorescence microscopy (DMI4000B, Leica, Mannheim, Germany).

### Cytosolic/Nuclear Fraction and RNA Florescent *in situ* Hybridization (RNA FISH)

The cellular fraction was isolated to locate the sublocation of ZEB1-AS1. Briefly, 10^7^ cells were harvested, resuspended in 1 ml of ice-cold RNase-free PBS, 1 ml of buffer C1 (1.28 M Sucrose, 40 mM Tris-HCl, pH 7.5, 20 mM MgCl_2_, 4% Triton X-100) and 3 ml of RNase-free water, and incubated for 15 min on ice. The cells were then centrifuged for 15 min at 3,000 × *g*, and the supernatant containing the cytoplasmic constituents and the nuclear pellet were retained for RNA extraction. GAPDH was set as the positive control for cytoplasm, and U1 served as the positive controls for the nucleus.

For RNA FISH, HUVECs were seeded and fixed with 4% paraformaldehyde, treated with 0.5% Triton in PBS, followed by pre-hybridization. They were then hybridized with the biotin-labeled ZEB1-AS1 probe (5 μM) overnight at 42°C. The anti-biotin monoclonal antibody and Alexa Fluor^®^647-conjugated secondary antibody (Invitrogen, Shanghai, China) were used for detecting ZEB1-AS1. Cells were washed with PBS and then placed on cover slips with prolong gold antifade reagent containing DAPI (Cell Signaling Technology, Cambridge, MA, United States). Stained cells were observed under an Olympus microscope supporting a Hamamatsu 1394 ORCA-ERA video camera and the images were stored using Slidebook Digital Microscopy Software (Intelligent Imaging Innovations). The ZEB1-AS1 probes were synthesized by Sangon (Shanghai, China).

### RNA Pulldown

The RNA pulldown assay was performed using a Magnetic RNA-Protein Pull-down Kit (Thermo Scientific) according to manufacturer’s instructions. HUVECs (2 × 10^7^) were cross-linked for each hybridization reaction. The cell lysates were hybridized with a mixture of biotinylated DNA probes for 4 h at 37°C. The binding proteins were separated by electrophoresis.

### RIP Assay and ChIP Assay

The RNA immunoprecipitation (RIP) was performed using the EZ-Magna RIP kit (Millipore, Burlington, MA, United States) according to the manufacturer’s instructions. Briefly, 10^7^ cells were lysed with RIP lysis buffer using one freeze-thaw cycle. Cell extracts were co-immunoprecipitated with anti-LRPPRC (ab205022, Abcam, Cambridge, MA, United States) antibody and the retrieved RNA was subjected to qRT-PCR analysis. IgG antibody (EMD Millipore, cat. no. 12–371) was used as a negative control. For qRT-PCR analysis, GAPDH was used as a non-specific control.

An EZ-Magna ChIP kit (Millipore) was used for the ChIP assay according to the manufacturer’s protocol. Briefly, cells were treated with formaldehyde and incubated for 10 min to generate DNA-protein cross-links. Cell lysates were then sonicated to generate chromatin fragments of 200–300 bp and immunoprecipitated with p53 antibody (Abcam, cat. no. ab131442) or IgG antibody (EMD Millipore, cat. no. 12–371). RNA was recovered and analyzed by qPCR.

### Immunofluorescence Assay

HUVECs were fixed in 4% formaldehyde for 15 min and then washed with PBS. The fixed cells were treated with pepsin and dehydrated through ethanol, and further permeabilized in Triton X100 (Sigma-Aldrich) for 20 min. Goat serum was used for blocked, and then cells were incubated with anti-NOD2 antibody (Abcam, ab36836, 1:500, Cambridge, MA, United States) for overnight at 4°C, and then incubated with the appropriate rhodamine-conjugated secondary antibody for 1 h at room temperature. The cells were then washed and incubated with DAPI (Invitrogen) for nuclear staining. The cells were visualized for immunofluorescence with a fluorescence microscopy (DMI4000B, Leica).

### Western Blots and Antibodies

RIPA buffer (Sigma-Aldrich) was used to lyse the cells to obtain total protein lysates. Protein concentration was measured using the BCA method (Sigma-Aldrich). The quantified protein (25 μg) was transferred onto polyvinylidene fluoride (PVDF) membranes (Sigma-Aldrich) following SDS-PAGE gel electrophoresis. Then, the membrane was blocked with 5% non-fat dry milk in tri-buffered saline plus Tween (TBS-T) buffer for 2 h at room temperature and incubated with anti-LRPPRC antibody (1:1000, ab205022, Abcam), anti-NOD2 antibody (1:1000, ab36836, Abcam) or anti-GAPDH antibody (Invitrogen, cat. no. PA1-987) at 4°C overnight, followed by Horseradish peroxidase-conjugated (HRP) secondary antibody (1:5000, Abcam, cat. no. ab7090) at room temperature for 1 h.

### Statistical Analysis

All *in vitro* experiments were performed using triple parallel samples and repeated triple times. Kolmogorov–Smirnov test was applied for data analysis with the distribution of each group samples. Data were presented as median (interquartile range). Mann–Whitney *U*-test was used for the comparison of datasets containing two groups. The Kruskal–Wallis test (*post hoc* Mann–Whitney *U*-test with Bonferroni’s) was used for analyzing statistical difference among multiple groups. A two-sided *P* < 0.05 was considered as statistical significance. Statistical analysis was performed using Prism 5 (GraphPad Software Inc., San Diego, CA, United States).

## Results

### Knockdown of lncRNA ZEB1-AS1 Alleviates ox-LDL-Induced Cell Injury in HUVECs

**FIGURE 1:**
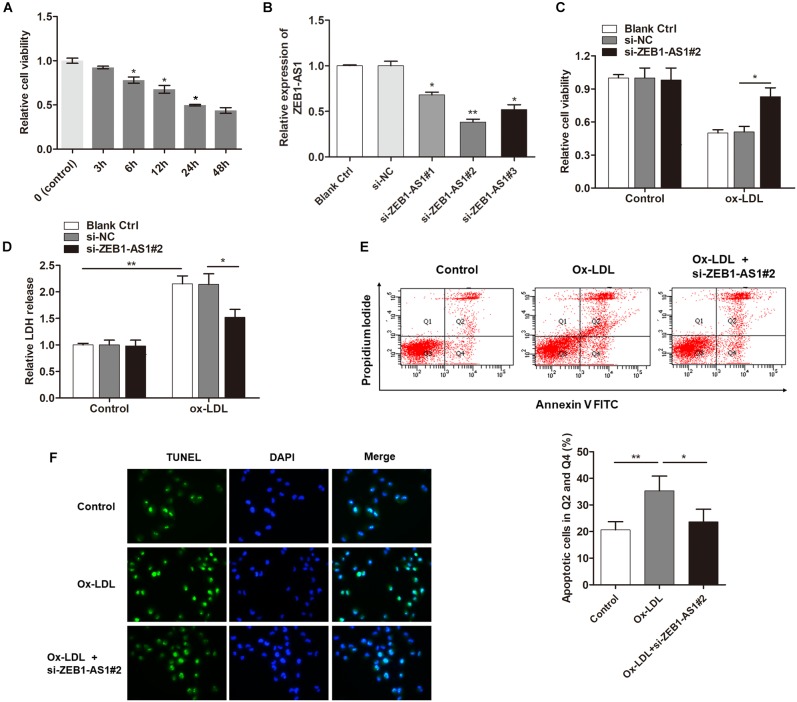
Silence of lncRNA ZEB1-AS1 abrogates oxidized-low density lipoprotein (ox-LDL)-induced cell injury in HUVECs. **(A)** HUVECs were treated with 100 μg/ml ox-LDL for respective time durations. MTT assay showed that ox-LDL treatment decreased cell viability, ^∗^*P* < 0.05 (Mann–Whitney *U*-test) was used for analyzing statistical difference among multiple groups compared to previous group. **(B)** qRT-PCR showed that ZEB1-AS1 was silenced by transfection of specific ZEB1-AS1 siRNAs (si-ZEB1-AS1#1, si-ZEB1-AS1#2, and si-ZEB1-AS1#3), ^∗^*P* < 0.05, ^∗∗^*P* < 0.01 (Kruskal–Wallis test) compared to si-NC and blank control cell group (without transfection). **(C,D)** HUVECs (treated with ox-LDL or not) were transfected with si-NC or si-ZEB1-AS1#2. Cell viability **(C)** and LDH release **(D)** were detected were measured by MTT assay and ELISA, respectively, ^∗^*P* < 0.05 (Mann–Whitney *U*-test), ^∗∗^*P* < 0.01 (Kruskal–Wallis test). **(E)** HUVECs apoptosis was detected by using flow cytometry after treatment with ox-LDL and/or knockdown of ZEB1-AS1 for 24 h. The data showed that ZEB1-AS1 knockdown reversed the apoptosis induced by ox-LDL treatment. ^∗^*P* < 0.05, ^∗∗^*P* < 0.01 (Mann–Whitney *U*-test). **(F)** Nuclear apoptosis was evaluated by using TUNEL assay in HUVECs treated with ox-LDL and/or si-ZEB1-AS1#2. ZEB1-AS1 knockdown reversed the apoptosis induced by ox-LDL treatment.

To investigate the functional role of ZEB1-AS1 in ox-LDL-induced HUVECs injury, we treated the HUVECs with 100 μg/ml ox-LDL. As shown in Figure 1A, ox-LDL treatment significantly suppressed cell viability of HUVECs at a time-dependent manner. Then, we characterized lncRNA ZEB1-AS1. 5- and 3-RACE assay was employed to determine the transcriptional start and termination sites of ZEB1-AS1. The full length of ZEB1-AS1 was shown to be of 2503 nt containing 1 exon, and located at Chr10: 31316528–31319030. We silenced ZEB1-AS1 in HUVECs by using specific silencing oligonucleotides (Figure 1B), and si-ZEB1-AS1#2 was used for further experiments. Knockdown of ZEB1-AS1 showed little effect on cell viability under normal condition and significantly increased cell viability when HUVECs was treated with ox-LDL (Figure 1C). Moreover, ox-LDL treatment (24 h) increased the release of LDH, however, this effect was dramatically reversed by knockdown of ZEB1-AS1 (Figure 1D). Flow cytometry analysis revealed that ox-LDL treatment increased HUVECs apoptosis, and ZEB1-AS1 silencing reversed this effect (Figure 1E). Similarly, TUNEL assay showed that knockdown of ZEB1-AS1 suppressed the ox-LDL-induced cell death (Figure 1F). Together, our results indicate that knockdown of ZEB1-AS1 could effectively abrogate the ox-LDL-induced HUVECs injury.

### ox-LDL Upregulates lncRNA ZEB1-AS1 Expression via Modulating p53 Modification at ZEB1-AS1 Promoter Region

**FIGURE 2:**
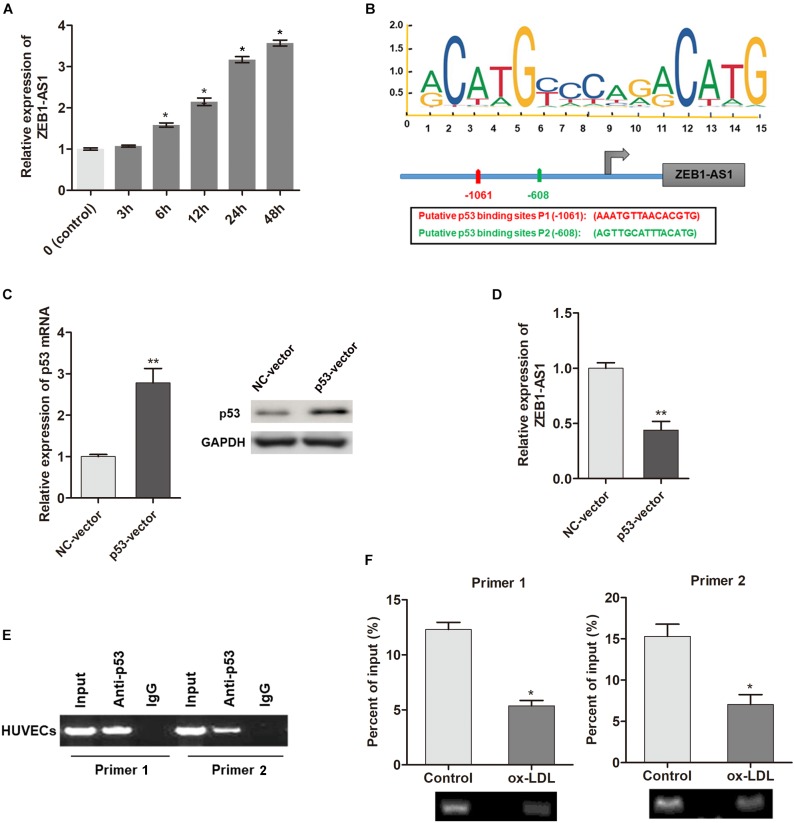
ox-LDL decreased p53 binding at ZEB1-AS1 promoter region, thereby activating the transcription of ZEB1-AS1. **(A)** The relative expression of ZEB1-AS1 was detected by qRT-PCR in HUVECs under normal control or ox-LDL condition, and ox-LDL treatment increased ZEB1-AS1 expression in a time-dependent manner. ^∗^*P* < 0.05 (Mann–Whitney *U*-test) compared to previous group. **(B)** Two binding sites of p53 (–1149, –534) at ZEB1-AS1 promoter were predicted by using online JASPAR software. **(C)** p53 was overexpressed in HUVECs by transfection of specific overexpression vector, ^∗∗^*P* < 0.01 (Mann–Whitney *U*-test). **(D)** Relative expression of ZEB1-AS1 was measured via qRT-PCR in cells overexpressed p53, ^∗∗^*P* < 0.01 (Mann–Whitney *U*-test). **(E)** ChIP assay was performed by using p53 antibody and two primers to detect the immunoprecipitations. **(F)** The enrichment of p53 at ZEB1-AS1 promoter region was evaluated via ChIP assay in cells treated with ox-LDL or not. The enrichment was significantly suppressed by ox-LDL treatment, ^∗^*P* < 0.05 (Mann–Whitney *U*-test).

To investigate the underlying mechanism by which ZEB1-AS1 mediates ox-LDL-induced cell injury, we determined the regulation of ZEB1-AS1 expression by ox-LDL. When HUVECs were treated with 100 μg/ml ox-LDL, we found that ZEB1-AS1 expression was increased in a time-dependent manner (Figure 2A). To verify how ox-LDL regulates ZEB1-AS1 expression, we analyzed the promoter region of ZEB1-AS1 by using the online transcription factor prediction software, JASPAR. Two potential binding sites of p53 were verified at the promoter region of ZEB1-AS1 (Figure 2B). Then, p53 was overexpressed in HUVECs by transfection of specific p53 overexpression vector (Figure 2C). Overexpression of p53 significantly inhibited ZEB1-AS1 expression (Figure 2D), suggesting that p53 suppresses the transcription of ZEB1-AS1 gene. To prove the direct interaction between p53 and ZEB1-AS1, we performed ChIP experiment by using specific primers. As shown in Figure 2E, the antibody directed against p53 immunoprecipitated the DNA fragments containing the potential binding sites of p53 at ZEB1-AS1 promoter region. Moreover, treatment with ox-LDL significantly decreased the enrichment levels of p53 at ZEB1-AS1 promoter region (Figure 2F). Therefore, our results demonstrated that ox-LDL decreased the direct binding of p53 to the ZEB1-AS1 promoter region, which relieved the p53-induced suppressive effect on ZEB1-AS1 gene transcription.

### ZEB1-AS1 Regulates ox-LDL-Induced ECs Injury via the Upregulation of NOD2 Level

**FIGURE 3:**
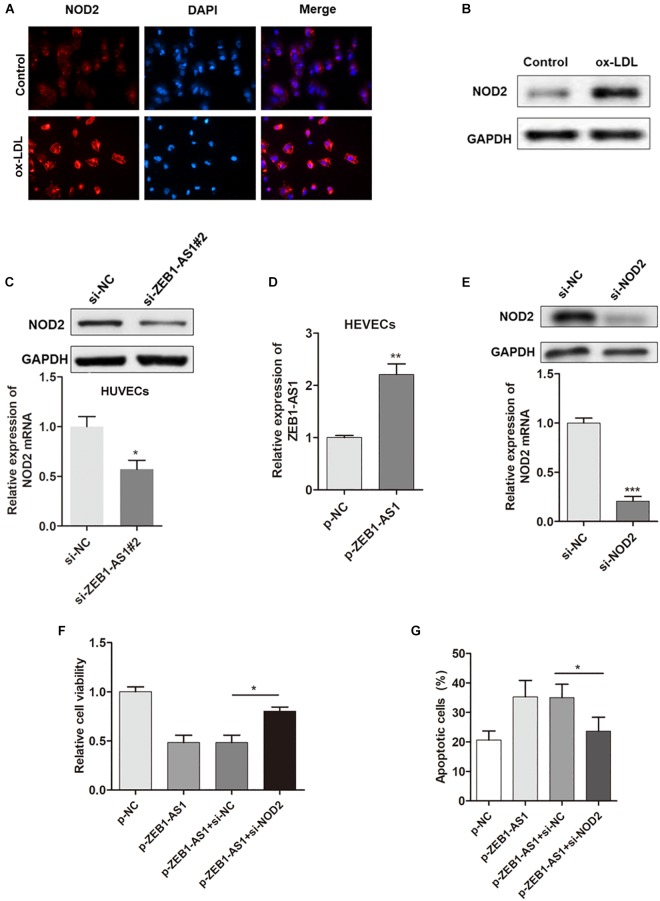
NOD2 was verified as a functional target of ZEB1-AS1 in ox-LDL-induced ECs injury. **(A)** The expression of NOD2 protein in HUVECs under normal or ox-LDL condition was assessed by using immunofluorescence. ox-LDL significantly increased NOD2 expression, ^∗^*P* < 0.05 (Mann–Whitney *U*-test). **(B)** Western blot analysis showed that ox-LDL treatment decreased NOD2 protein level. **(C)** The expression of NOD2 mRNA (left panel) and protein (right panel) was downregulated by ZEB1-AS1 knockdown, ^∗^*P* < 0.05 (Mann–Whitney *U*-test). **(D)** ZEB1-AS1 was overexpressed in HUVECs by transfection of ZEB1-AS1 overexpression plasmid, ^∗∗^*P* < 0.01 (Mann–Whitney *U*-test). **(E)** NOD2 was silenced in HUVECs at both transcript (left panel) and protein (right panel) levels by transfection of specific siRNA, ^∗∗∗^*P* < 0.001 (Mann–Whitney *U*-test). **(F,G)** Cell viability **(F)** and apoptosis **(G)** were evaluated by MTT assay and flow cytometry assay, respectively. Knockdown of NOD2 attenuated the functional effect induced by ZEB1-AS1 overexpression, ^∗^*P* < 0.05 (Mann–Whitney *U*-test).

Our previous study showed that NOD2 played a critical role in ECs injury, thus we assume ZEB1-AS1 may contribute to the ox-LDL-induced ECs injury through targeting NOD2. Immunofluorescence showed that ox-LDL treatment significantly increased the expression of NOD2 (Figure 3A). Western blot analysis further confirmed the upregulated NOD2 protein upon ox-LDL treatment (Figure 3B). Gain- or loss- functional assays showed that knockdown of ZEB1-AS1 significantly decreased NOD2 level at both transcript and protein levels (Figure 3C). Then, we tested the functional role of NOD2 in ox-LDL/ZEB1-AS1-mediated ECs injury by overexpression of ZEB1-AS1 and knockdown of NOD2 (Figure 3D,E). As shown in Figure 3F, enhanced expression of ZEB1-AS1 decreased HUVECs viability under ox-LDL treatment, however, this effect was significantly attenuated by NOD2 knockdown. Similarly, knockdown of NOD2 reversed the ZEB1-AS1-induced HUVECs apoptosis according to flow cytometry analysis (Figure 3G). These data showed that ZEB1-AS1 regulates ECs injury via the upregulation of NOD2.

### ZEB1-AS1 Directly Interacts With LRPPRC to Play Key Roles in ECs Injury

**FIGURE 4:**
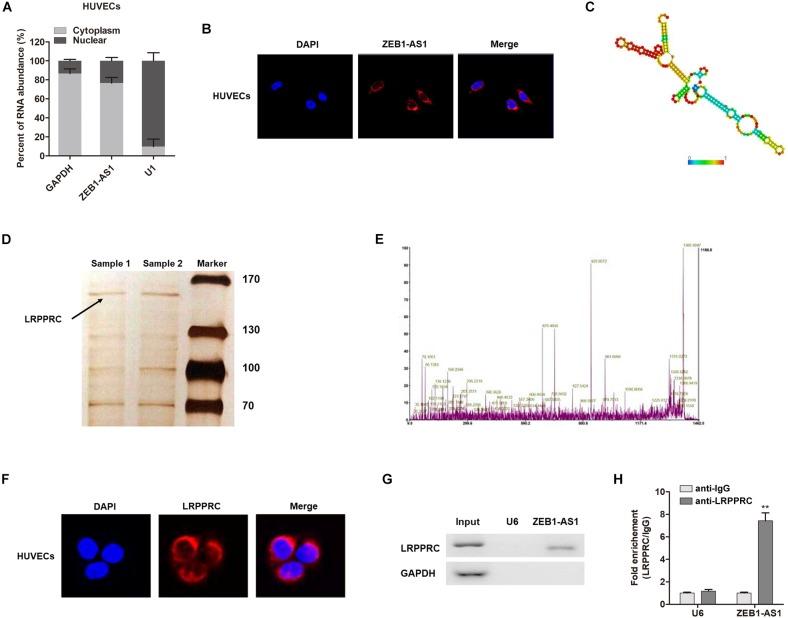
ZEB1-AS1 directly interacts with LRPPRC in ECs. **(A)**. Nuclear fraction experiment and qRT-PCR detected the abundance of ZEB1-AS1 in the nucleus and cytoplasm. GAPDH was set as the positive control for cytoplasm, and U1 served as the positive controls for the nucleus. **(B)** The subcellular distribution of ZEB1-AS1 was visualized by RNA Fluorescent *in situ* hybridization (FISH) in HUVECs. ZEB1-AS1 was mainly distributed in cytoplasm of HUVECs. **(C)**. Prediction of 421–630 nt ZEB1-AS1 structure was based on minimum free energy (MFE) and partition function (http://rna.tbi.univie.ac.at/). **(D)** RNA pulldown assay was performed using ZEB1-AS1 incubated with cytoplasm extracts of HUVECs, followed by silver staining. An arrow indicates LRPPRC. **(E)** Verification of LRPPRC protein by using mass spectrometry. **(F)** The subcellular distribution of LRPPRC (cytoplasm) was visualized by immunofluorescence in HUVECs. **(G)** The interaction between ZEB1-AS1 and LRPPRC was confirmed by RNA pulldown and western blotting. GAPDH served as negative controls. **(H)** RIP was performed using anti-LRPPRC and control IgG antibodies, followed by qRT-PCR to examine the enrichment of ZEB1-AS1 and U6. U6 served as negative controls. ^∗∗^*P* < 0.01 compared respective IgG group (Mann–Whitney *U*-test).

The subcellular localization of a lncRNA is associated closely with its biological mechanism. Cellular fractionation assays and RNA florescent *in situ* hybridization (RNA-FISH) showed that ZEB1-AS1 was distributed mainly in the cytoplasm in ECs cells (Figure 4A,B). lncRNAs located in the cytoplasm are usually associated with post-transcriptional regulation (Chen et al., 2017; Lu et al., 2018). To investigate the underlying mechanism by which ZEB1-AS1 regulates NOD2, we determined the second structure of ZEB1-AS1. Based on minimum free energy (MFE) and partition function^1^, we predicted that nucleotides 421–630 nt in the ZEB1-AS1 transcript, formed a stem-loop structure (Figure 4C), which is critical for the physical interaction with proteins. To identify ZEB1-AS1-interacting proteins in HUVECs, we performed RNA pulldown assays using *in vitro* transcribed biotinylated ZEB1-AS1 followed by mass spectrometry. A list of proteins were identified (Table 2). One overtly differential band (∼150 kDa) appeared after silver staining and was identified as leucine rich pentatricopeptide repeat containing (LRPPRC) by mass spectrometry (Figure 4D,E). Consistent with ZEB1-AS1 localization, LRPPRC was also distributed mainly in the cytoplasm in HUVECs (Figure 4F). We further confirmed the special interaction between ZEB1-AS1 and LRPPRC using RNA pull down and RNA immunoprecipitation (RIP) (Figure 4G,H). Our results suggest that ZEB1-AS1 directly interacts with LRPPRC in HUVECs.

**Table 2:** Mass spectrometry analysis of the proteins pulled down by ZEB1-AS1 in HUVECs.

Number	Protein name	Beads	ZEB1-AS1 cover percent
1	LRPPRC	0	42.33%
2	STT3B	0	27.62%
3	AKAP8	0	24.78%
4	RA1L2	0	20.38%
5	LAS1L	0	20.14%
6	DDX17	0	16.15%
7	PCH2	0	8.90%
8	UBP10	0	5.87%
9	PTBP1	0	2.36%
10	DPM1	0	1.87%

*Beads, spectral counts of proteins in beads only group; ZEB1-AS1 cover percent, percent of spectral counts of proteins in ZEB1-AS1 group.*

### ZEB1-AS1 Enhances mRNA Stability of NOD2 via Recruiting LRPPRC

**FIGURE 5:**
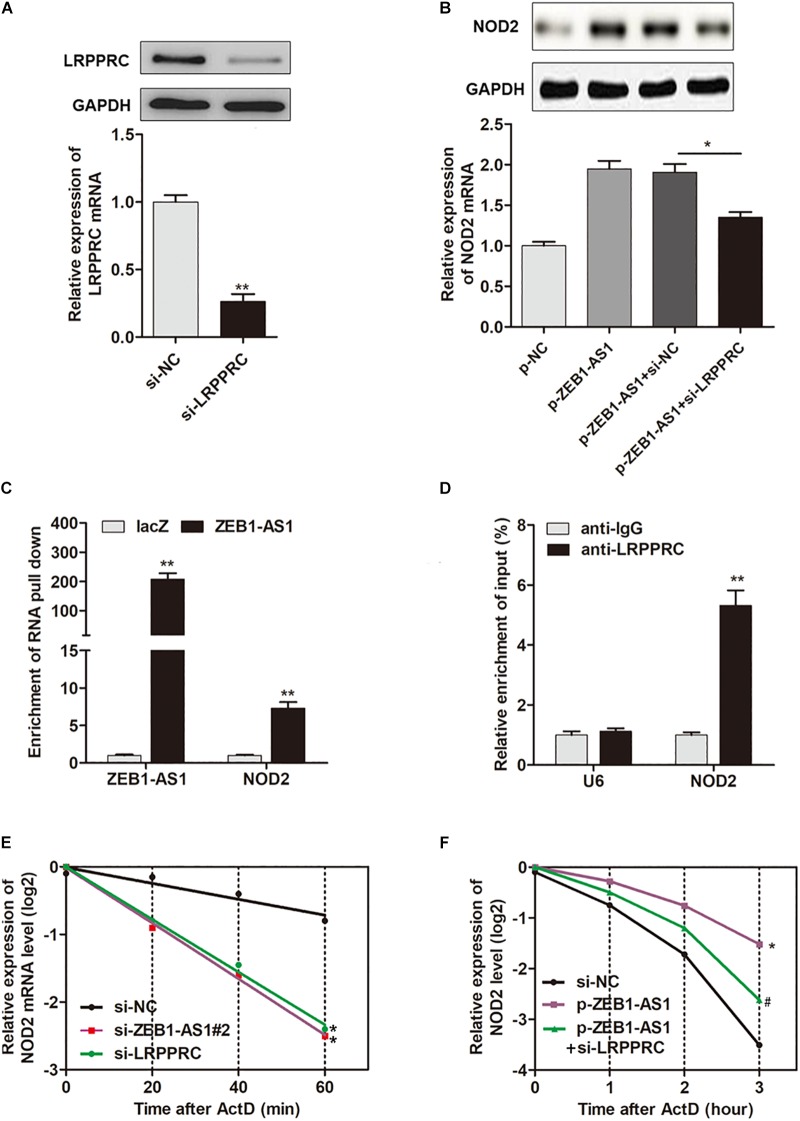
ZEB1-AS1 regulates mRNA stability of NOD2 via recruiting LRPPRC. **(A)** LRPPRC was silenced by specific siRNAs at both transcript (down panel) and protein (upper panel) levels, ^∗∗^*P* < 0.01 (Mann–Whitney *U*-test). **(B)** NOD2 expression was detected in HUVECs overexpressed with ZEB1-AS1 and/or silenced with LRPPRC, ^∗^*P* < 0.05 (Mann–Whitney *U*-test). **(C)** HUVECs lysates were incubated with *in vitro* synthesized, biotin-labeled control LacZ DNA probes or DNA probes against ZEB1-AS1 for the biotinylated oligonucleotide pulldown assay. The precipitates from the pulldown were analyzed by qRT-PCR to detect the interacting mRNAs. ^∗∗^*P* < 0.01 (Mann–Whitney *U*-test) compared to respective LacZ probes. **(D)** RIP was performed using anti-LRPPRC and control IgG antibodies, followed by qRT-PCR to examine the enrichment of NOD2 and U6. U6 served as negative controls. ^∗∗^*P* < 0.01 (Mann–Whitney *U*-test) compared to respective IgG controls. **(E)** HUVECs expressing si-ZEB1-AS1#2 or si-LRPPRC were treated with actinomycin D (5 μg/mL) for the indicated periods of time. ^∗^*P* < 0.05 (Mann–Whitney *U*-test) compared to si-NC group **(F)**. HUVECs expressing si-NC, or p-ZEB1-AS1, or p-ZEB1-AS1 + si-LRPPRC were treated with actinomycin D (5 μg/mL) for the indicated periods of time. Total RNA was purified and then analyzed using qRT-PCR to examine the mRNA half-life of NOD2. ^∗^*P* < 0.05 (Mann–Whitney *U*-test) compared to si-NC group, and ^#^*P* < 0.05 (Mann–Whitney *U*-test) compared to p-ZEB1-AS1 group.

It is reported that LRPPRC play key roles in promoting mRNA stability (Siira et al., 2017; Chen et al., 2019). Hence, we assume that ZEB1-AS1 may guide LRPPRC to enhance the stabilization of NOD2 mRNA, thereby contributes to the ox-LDL-induced ECs injury. To prove this hypothesis, we silenced LRPPRC in HUVECs (Figure 5A) and found that LRPPRC knockdown eliminated the ZEB1-AS1-mediated upregulation of NOD2 expression in both mRNA and protein levels (Figure 5B). To determine whether ZEB1-AS1 directly interacts with NOD2 mRNA, we performed a biotinylated oligonucleotide pulldown assay. We found that endogenous NOD2 mRNA was co-precipitated with ZEB1-AS1 in HUVECs (Figure 5C). In addition, RIP assay revealed that NOD2 mRNA was enriched in LRPPRC precipitates, indicating that ZEB1-AS1 and LRPPRC could directly interact with NOD2 (Figure 5D). Furthermore, we treated HUVECs with actinomycin D (ActD), which allowed us to measure the decay of pre-existing mRNA. The data showed that knockdown of ZEB1-AS1 or LRPPRC resulted in a decreased half-life of NOD2 mRNA in HUVECs (Figure 5E), whereas overexpression of ZEB1-AS1 increased its half-life (Figure 5F). Moreover, knockdown of LRPPRC eliminated the increased half-life induced by overexpression of ZEB1-AS1 (Figure 5F). Taken together, our data indicated that ZEB1-AS1 guides LRPPRC to stabilize NOD2 mRNAs, contributing to its increased expression in HUVECs.

### ZEB1-AS1 Modulates ox-LDL-Induced ECs Injury by Stabilization of NOD2 mRNA

**FIGURE 6:**
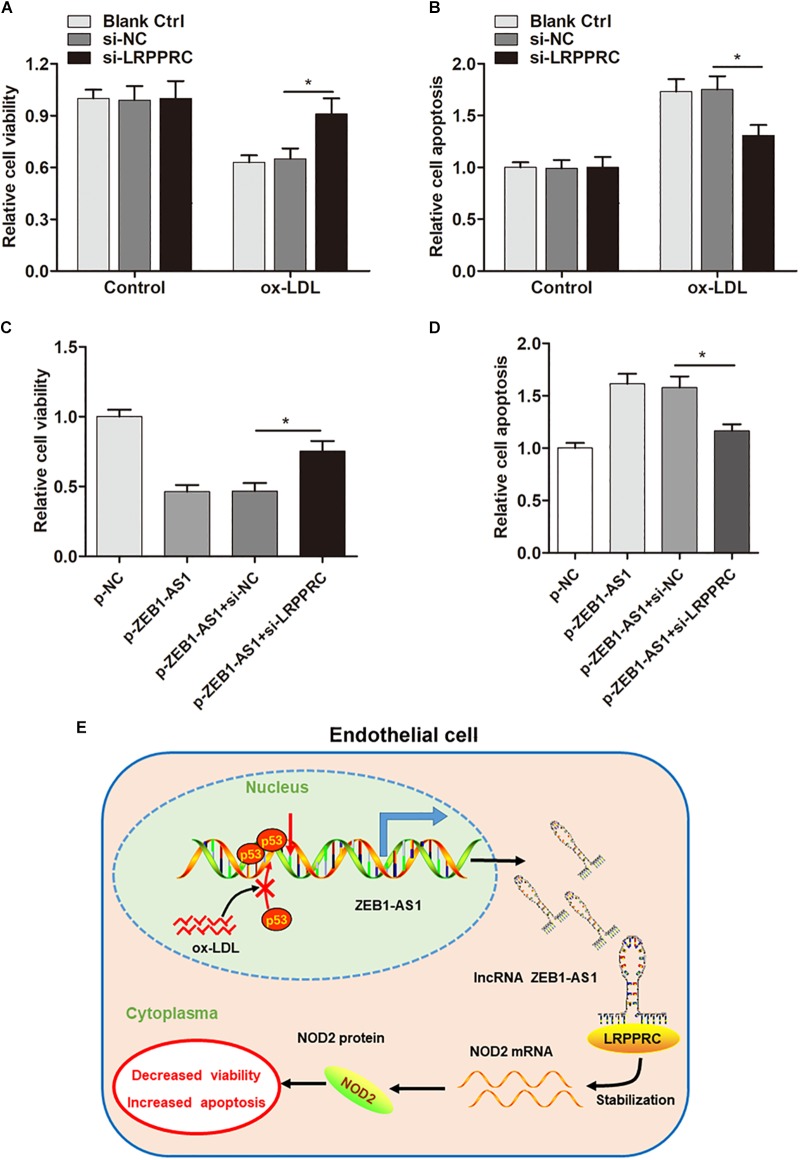
ZEB1-AS1 modulates ox-LDL-induced ECs injury by stabilization of NOD2 mRNA. **(A,B)** HUVECs (treated with ox-LDL or not) were transfected with si-NC or si-LRPPRC. Cell viability **(A)** and LDH release **(B)** were detected were measured by MTT assay and flow cytometry, respectively, ^∗^*P* < 0.05 (Mann–Whitney *U*-test). **(C,D)** Cell viability **(C)** and apoptosis **(D)** were evaluated by MTT assay and flow cytometry assay, respectively, ^∗^*P* < 0.05 (Mann–Whitney *U*-test). **(E)** A scheme of the proposed mechanisms. ox-LDL treatment increases ZEB1-AS1 expression by sequestering p53 from binding to the promoter region of ZEB1-AS1 gene, which activates the transcription and upregulates ZEB1-AS1 level in HUVECs. Overexpression of ZEB1-AS1 upregulates NOD2 level by guiding LRPPRC to stabilize its mRNA, resulting in decreased viability and increased apoptosis of ECs.

After having validated the direct interaction between ZEB1-AS1, LRPPRC, and NOD2, we sought to verify whether ZEB1- AS1 mediates ox-LDL-induced HUVECs injury by stabilization of NOD2. As expected, knockdown of LRPPRC increased cell viability and decreased cell apoptosis (Figure 6A,B). Moreover, silence of LRPPRC abrogated the effect on HUVECs injury induced by ZEB1-AS1 overexpression (Figure 6C,D). These results clearly revealed that ZEB1-AS1 modulates ox-LDL-induced ECs injury by stabilization of NOD2 mRNA.

Overall, our study demonstrated that ox-LDL could upregulate ZEB1-AS1 expression level in ECs, which thereby promoted ECs injury by post-transcriptionally stabilization of NOD2 mRNA in an LRPPRC-dependent manner (Figure 6E), suggesting that ZEB1-AS1 may be a promising therapeutic target for patients with AS or ECs injury–related diseases.

## Discussion

Numerous studies in recent years have helped to gain a better understanding of the molecular mechanisms during ECs injury and AS progression (Sumiyoshi and Asada, 1988; Pech-Amsellem et al., 1996; Li et al., 2017). However, the specific regulatory model is still largely unknown, and it is of much importance to discover new molecular signatures which may be useful for AS prevention and therapy. Our previous study revealed the functional role of lncRNA XIST in ox-LDL-induced cell injury (Xu et al., 2018). However, the roles of lncRNAs in ox-LDL-induced ECs injury is still largely unknown. In this study, we found that another lncRNA, ZEB1-AS1, mediated ox-LDL-induced ECs injury. Mechanistically, ox-LDL upregulated ZEB1-AS1 level via decreasing p53 modification at the promoter region of ZEB1-AS1 gene. ZEB1-AS1 could enhance the expression of NOD2 by post-transcriptionally stabilization of NOD2 mRNA dependent on LRPPRC.

lncRNAs are reported to regulate biological functions via diverse mechanism: guider; decoy; scaffold effect on DNA, RNA or protein; and post-transcriptional effects (Ulitsky and Bartel, 2013). The expression of ZEB1-AS1 has been reported and was identified as an oncogene in several cancer types, such as gastric cancer (Xu et al., 2017), prostate cancer (Su et al., 2017), colorectal cancer (Xiong et al., 2018), and glioma (Lv et al., 2016). In addition, one study by Wang et al. (2018) demonstrated that lncRNA ZEB1-AS1 could promote renal fibrosis in diabetic nephropathy. However, the functional role of ZEB1-AS1 in AS or other ECs injury-related disease is not reported. Li et al. (2017) reported the length of ZEB1-AS1 was 2,449 bp, lacking more in-depth data (Caruso et al., 2014). The discrepancies above might be explained by different cell lines used in different studies or different splicing variants of ZEB1-AS1. To the best of our knowledge, this is the first study to systematically evaluate the role of ZEB1-AS1 in ECs injury and the underlying regulatory mechanism. We found that ZEB1-AS1 is critical for ox-LDL-induced cell injury and may serve as a therapeutic target.

We investigated the underlying mechanism by which ox-LDL regulates ZEB1-AS1 expression during ECs injury. Recent studies revealed that activation or inhibition of transcription via the various modifications at gene promoters is the major factor that controls gene expression in a temporal and spatial manner, resulting in the establishment and maintenance of biological changes (Piunti and Pasini, 2011). Since ZEB1-AS1 is reported to be suppressed by p53 (Wang et al., 2018), we investigated whether ox-LDL influence the expression of ZEB1-AS1 by regulation of p53 binding. Luckily, we identified that p53 was associated with ZEB1-AS1, resulting a suppression of ZEB1-AS1 transcription, which is consistent with the previous report. More importantly, treatment with ox-LDL could dramatically decrease the binding level of p53, which finally released the activation of ZEB1-AS1.

The subcellular localization of lncRNA is associated closely with its biological mechanism. Our results showed that ZEB1-AS1 was distributed mainly in the cytoplasm in ECs, indicating it may regulate ECs injury at the post-transcriptional level. Previously, we reported that NOD2 is critical for ECs cell apoptosis and AS progression (Liu et al., 2013; Yuan et al., 2013; Kim, 2015). NOD2, a major pattern recognition receptor integrating ER stress and inflammation, is essential for antimicrobial innate immunity and tissue homeostasis (Liu et al., 2013). Interactions between pattern recognition receptors shape innate immune responses to particular classes of pathogens, such as ECs injury and AS (Oh et al., 2005), which may account for the important function of NOD2 in ECs injury. Our study further verified the regulation of NOD2 by ZEB1-AS1 to affect the initiation and progression of ECs injury. More importantly, ZEB1-AS1 directly bind to LRPPRC to influence the mRNA stability of NOD2. LRPPRC, an RNA binding protein, regulates mRNA stability and polyadenylation mainly in mitochondria, but also in the cytoplasm and nucleus (Tsuchiya et al., 2004; Chujo et al., 2012; Ruzzenente et al., 2012; Volpon et al., 2017). We found that both ZEB1-AS1 and LRPPRC were distributed mainly in the cytoplasm in HUVECs. Knockdown ZEB1-AS1 or LRPPRC resulted in a decrease in the half-life of NOD2 in HUVECs. Overall, we identified a novel mechanism by which ZEB1-AS1 guides LRPPRC to stabilize NOD2 mRNA, which increases NOD2 protein level and enhances ox-LDL-induced ECs injury.

Recently, small molecules designed to target the folded structure of pathologic non-coding RNA have shown significant anti-disease effects *in vivo* by selectively modulating non-coding RNAs in lesion cells, such as cancer (Velagapudi et al., 2016). Additionally, siRNA or locked nucleic acids can serve as therapeutic agents that specifically target lncRNAs *in vivo* (Disney and Angelbello, 2016). In the future, inhibition of ECs injury and AS progression via small molecules that specifically target ZEB1-AS1 might represent potential therapeutic methods.

## Conclusion

We report the discovery that ZEB1-AS1 functionally participates in ox-LDL-induced ECs injury via LRPPRC-mediated stabilization of NOD2. Uncovering the precise role of ZEB1-AS1/LRPPRC/NOD2 pathway in the progression of ox-LDL-induced ECs death and AS will not only increase our knowledge of ox-LDL-induced AS, but also enable the development of novel therapeutic strategies to overcome oxidation product-induced diseases.

## Author Contributions

XX and CL designed and mainly did the study. CM, ZD, and YD helped and did the study.

## Conflict of Interest Statement

The authors declare that the research was conducted in the absence of any commercial or financial relationships that could be construed as a potential conflict of interest.

## Footnotes

1 http://rna.tbi.univie.ac.at/
